# Psychometric analysis of stress, anxiety and depression in patients with recurrent aphthous Stomatitis-A cross-sectional survey based study

**DOI:** 10.4317/jced.55012

**Published:** 2018-11-01

**Authors:** Ashwini Dhopte, Giridhar Naidu, Ramanpal Singh-Makkad, Ravleen Nagi, Hiroj Bagde, Supreet Jain

**Affiliations:** 1 Post Graduate Student, Department of Oral Medicine and Radiology; 2 Professor and Head of the Department, Department of Oral Medicine and Radiology; 3 Associate Professor, Department of Oral Medicine and Radiology; 4 Associate Professor, Department of Oral Medicine and Radiology; 5 Assistant Professor, Department of Periodontology; 6 Assistant Professor, Department of Oral Medicine and Radiology

## Abstract

**Background:**

Recurrent Aphthous Stomatitis is a chronic inflammatory immune mediated condition associated with single or multiple, painful recurring ulcers of the oral mucosa. Psychological stress is a common trigger factor identified for the onset and progression of this condition. The study aimed to evaluate stress, anxiety and depression using the Hospital Anxiety and Depression scale and stress by the General Health Questionnaire in subjects with Recurrent Aphthous Stomatitis (RAS).

**Material and Methods:**

The study evaluated subjects with a history of Recurrent Aphthous Stomatitis and compared their psychological stress, anxiety and depression alteration to normal subjects. Seventy-five study subjects were divided into three groups; test group 1 (25 subjects) presenting with RAS, test group 2 (25 subjects) with a history of RAS but without oral ulcers, and 25 normal subjects with no history of RAS formed the control group. Chi square and student t test was used to determine the relationship between psychological variables and recurrent aphthous ulcers.

**Results:**

Comparison of stress, Anxiety and Depression between test group 1 and control group, test group 2 and control group was not found to be statistically significant (*p*< 0.05). Hence, differences were evident when individual questions were analyzed for stress anxiety and depression. Stress and depression were more prevalent in females, students and patients with a history of RAS.

**Conclusions:**

Higher depression and stress levels were evident in subjects with a history of RAS. Psychological stress is a utmost trigger factor for the initiation of recurrent ulcers. Overall, test group patients had a more psychological impact on the quality of life than the control group.

** Key words:**Anxiety, depression, General Health Questionnaire, Hospital Anxiety and Depression scale, Recurrent Aphthous Stomatitis, stress.

## Introduction

Recurrent aphthous stomatitis (RAS) or canker sore is painful and ulcerative disorder of the oral mucosa, affecting 5-25% of the general population characterized by recurrent, small, round or ovoid ulcers with circumscribed margins, erythematous halo and yellow or grayish floor, common in childhood or adolescence (1). The exact etiopathogenesis is still not clear, but evidence from the existing literature suggests focal immune dysfunction involving T cell mediated immunity as a key mechanism in the development of Recurrent Aphthous ulcer. Clinicians should also identify triggers or causative factors for proper management of this condition. Acute psychological disorders (e.g. Stress and anxiety) were found to be most commonly associated with the onset and progression of RAS as severe stress provokes immune-regulatory activity by increasing the number of leukocytes at sites of inflammation (2).

It is well recognized that the symptoms caused by recurrent aphthous stomatitis, such as pain during speaking, eating, and swallowing; discomfort; impairment in food and liquid intake; and problems in interpersonal relationships and self-esteem can deeply affect the oral health-related quality of life of patients (3). Oral health and psychological status has profound impact on people’s daily living, quality of life and overall well-being, therefore, the clinical status, psychological factors, dental needs and patient’s perceptions should be assessed (2). Taking above into consideration, this study was conducted to evaluate anxiety and depression using the Hospital Anxiety and Depression scale (4) and stress by General Health Questionnaire (5) in subjects presenting with Recurrent Aphthous Stomatitis and those having history of RAS but not presenting with an ulcer at present and were compared with controls.

## Material and Methods

The study enrolled Seventy-five subjects aged 15-50 years selected from the outpatient department of Oral Medicine and Radiology, New Horizon Dental College and Research Institute, Bilaspur, Chhattisgarh, India, after obtaining their informed consent. A Study was conducted from December 2015 to June 2017 and ethical clearance was obtained from the ethical committee of the institute. Patients presenting with one or more active ulcer (<48 hours duration) measuring no more than 10 mm in diameter were included and patients with artificial pace makers, malignant diseases, history of systemic disease i.e. HIV, anemia, mutational defects ,diabetes mellitus, etc or any condition that could predispose to oral ulceration, ulcers caused due to topical or systemic medications or currently under treatment with any topical medication or corticosteroids, pregnant or lactating women and chronic smokers were excluded from the study.

Seventy-five subjects were divided into three groups, test Group 1 (25 Subjects) presenting with RAS, test Group 2 (25 Subjects) with a history of RAS but not presenting with an ulcer at present, and 25 normal healthy adults with no history of RAS were selected as controls. Detailed case history was taken and were asked to fill General Health Questionnaire (GHQ) and Hospital Anxiety and Depression scale (HAD) questionnaire in English and Hindi language. Data gathered from completing questionnaires was scored and tabulated after it was entered into Microsoft excel spreadsheet and statistical analysis was done by using Statistical package of social science (SPSS session 21, SPSS Inc., Chicago, IL, USA). Chi square and student t test was used to determine the relationship between psychological variables and recurrent aphthous ulcers. *P* value <0.05 was considered to be statistically significant.

## Results

The mean age of the RAS patients in test group was 23.5 years and control group was 24.4 years. Out of 75 subjects, there were 33.3% males, and 66.7% females, with a male to female ratio of 1:2. Both RAS patients and controls reported comparable depression, anxiety, stress scores, that were statistically not significant (*p*>0.05). For RAS patients (test group 1), mean anxiety (8.04± 3.49), depression (5.20± 2.66) and GHQ stress (9.76± 4.52) scores were slightly higher than the control group where the scores were 7.04± 5.10, 3.96± 2.93 and 9.72± 5.0 respectively. The findings indicate that RAS patients had higher levels of anxiety, stress and depression than the control group as in  Table 1.

**Table 1:**
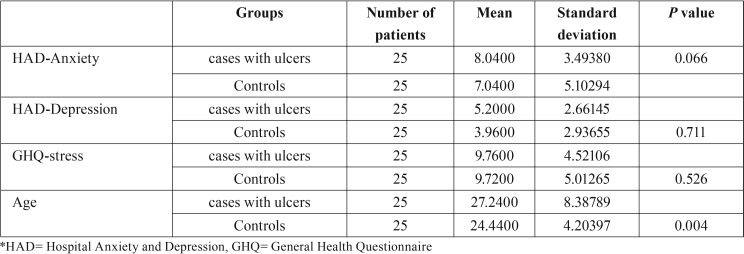
Comparison of psychological factors between test group 1 (cases with ulcers) and control group.

For patients with previous history of RAS (test group 2), mean GHQ stress (11.48± 5.66) and depression (5.36± 3.18) score were higher than the control group for which the scores were 9.72± 5.01 and 3.96± 2.93, respectively whereas mean anxiety scores for test group 2 of 6.12± 3.14 was found to be slightly less than the controls of 7.04± 5.10 as shown in  Table 2. This suggested higher levels of stress and depression in patients with history of RAS in comparison to controls that were slightly more anxious.

**Table 2:**
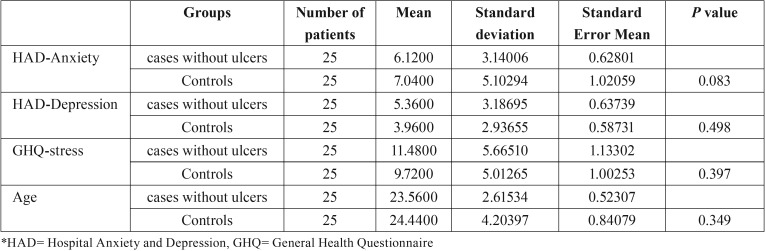
Comparison of psychological factors between test group 2 (cases with history of ulcer) and control group.

**Figure 1:**
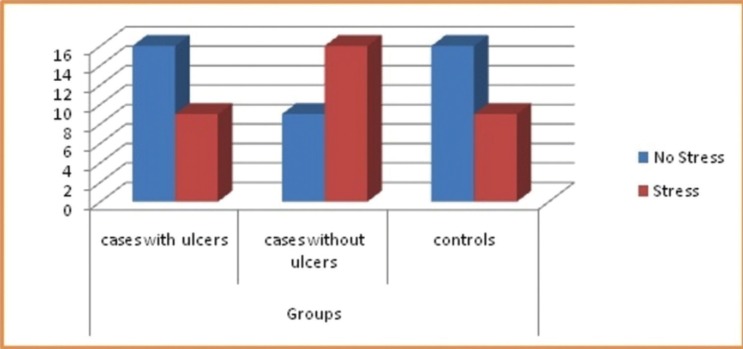
Distribution of subjects among group for Stress using scoring criteria.

**Figure 2:**
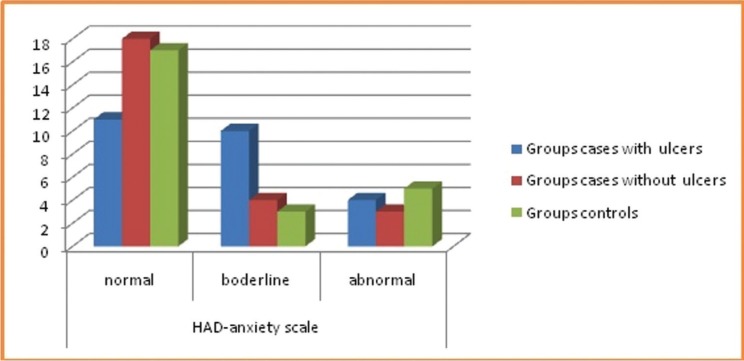
Distribution of subjects among groups for Anxiety using scoring criteria.

**Figure 3:**
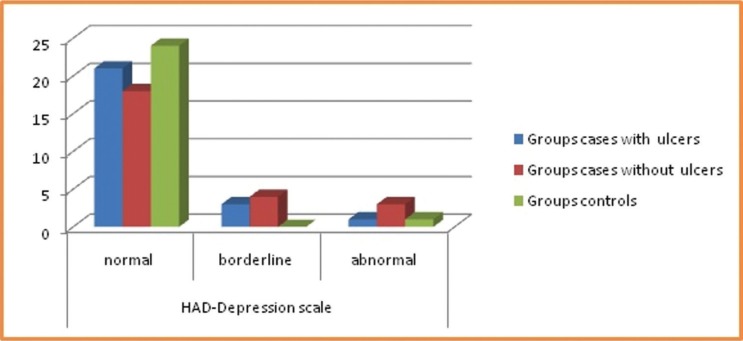
Distribution of subjects among groups for depression using scoring criteria.

The results obtained for the study regarding stress, anxiety and depression amongst both the groups had an impact on the overall health of the individual and also has triggering effect for RAS as shown in figures 1-3.

Results showed that mental stress had a larger effect on females than males and higher scores of stress, anxiety and depression were seen in students as compared to other profession and housewives. When a comparison was made between groups, there was no statistical significant difference noted in levels of anxiety, stress and depression, however, the differences in response became evident when individual questions were analyzed for stress, anxiety and depression as shown in  Table 3,  Table 4. Patients with previous history of ulcer had more stress and depression levels in comparison to controls. GHQ questionnaire responses demonstrated twice increase in stress levels for test groups than controls as most of them were not able to concentrate, we’re not able to take their own decisions, overcome difficulties, faced problems, lacked confidence and were unhappy. The psychological impact was also more in test group patients as they were tensed, worried and had a feeling of panic.

**Table 3:**
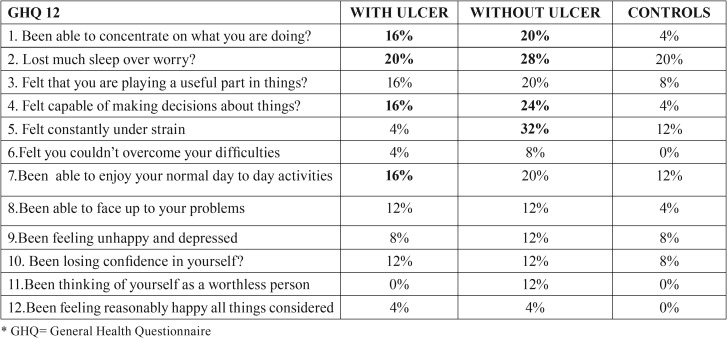
Comparison of responses among groups for psychological stress by GHQ questionnaire.

**Table 4:**
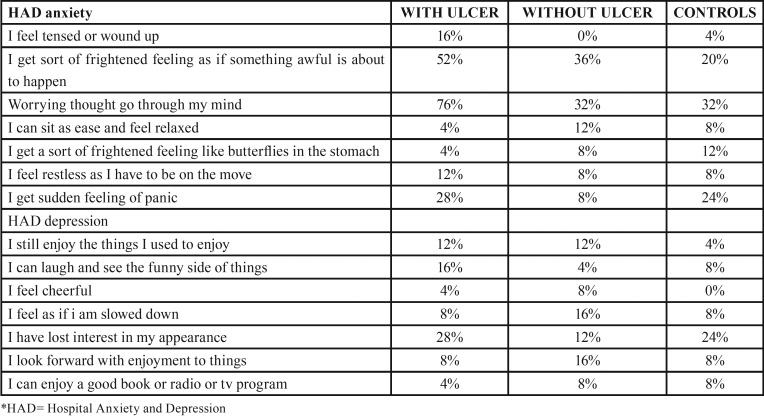
Comparison of responses among groups for anxiety and depression by Hospital anxiety and depression scale.

## Discussion

RAS are single or multiple painful ulcerations that usually occur on non-keratinized mucosa of the oral cavity and appear as yellowish white ulcerations, surrounded by an erythematous halo. It affects 20% of the general population with a predilection for females, commonly seen in children and young adults, and after a time tends to decrease both in severity and frequency. The exact etiology of RAS still remains unclear, and multiple factors have been considered in the exacerbation of RAS such as genetic predisposition, local trauma, allergy to certain food materials, vitamin deficiency, immunosuppression, smoking and psychiatric factors (stress and anxiety) (6). Psychological stress is considered as a common provoking or trigger factor in the occurrence and progression of RAS, but little documentation has been presented to substantiate this widely held assumption (7-9). This particular study was carried out to establish an association between RAS, stress, anxiety and depression by use of General Health Questionnaire, and Hospital anxiety and Depression scale. 25 cases of RAS patients were included in the present study with age range 16-55 years, the demographic data was similar to the study by Gallo *et al.* (10) in which the mean age of the RAS patients in test group was 23.5 years and control group 24.4 years. Out of 75 subjects, male patients accounted for 33.3%, and female patients accounted for 66.7%, with a male to female ratio of 1:2 in accordance with studies by Ship (11) Gallo *et al.* (10) that reported female predilection for RAS.

Life’s stress is a product of the changes that occur in one’s life that require adaptation, coping and social readjustment (12), and often it may disturb the psychic equilibrium, producing maladaptive patterns and possibly physical and mental disorders (13-15). Higher levels of anxiety were associated with recurrent ulcers which may be because of transient and episodic stressful situations that trigger certain mechanisms (such as transitory increase of salivary cortisol and stimulation of immune-regulatory activity by increasing the number of leukocytes at sites of inflammation) (7,14). In the present study, patients with RAS reported slightly higher levels of anxiety (8.04± 3.49) than controls (7.04± 5.10), and this association was in agreement with the studies by Buajeeb *et al.* (13) Gallo *et al.* (10) and Soto-Araya *et al.* (14) Depression levels for RAS patients (5.20± 2.66) were also higher when compared to control group (3.96± 2.93), but the difference was statistically not significant (0.711), this finding was similar to the results obtained by Mc Cartan *et al.* (7) and Soto -Araya *et al.* (14) in which no statistically significant relationship was demonstrated between depression and RAS.

When patients with RAS were evaluated by the psychometric test using GHQ score, it was observed that the mean GHQ score for test group 1 was 9.76± 4.52 which was comparable to control group 9.72± 5.01 (*p*=0.526) but higher levels were observed for test group 2 (11.48± 5.66, *p*=0.397), and the difference was not statistically significant, this shows that patients with ulcer history were having slightly more stress and those with RAS at present had similar stress levels as normal individuals. Contrary to above observation, Pedersen A (9), Andrews and Hall (16), Zwiri *et al.* (17) Sherman *et al.* (18) reported no association between RAS and stressful events or symptoms of anxiety, hence the authors concluded that the psychological factors were neither important, in the etiopathogenesis nor in the severity of the RAS.

## Conclusions

This study suggested impact of psychological factors such as anxiety, stress and depression on the quality of life of RAS patients. Although depression and anxiety levels were comparable to normal individuals, but psychological stress was found to be an important trigger factor for the initiation of recurrent ulcers. More survey based questionnaire studies or clinical trials should be encouraged for establishment of a significant relationship between psychological status and RAS.
